# Supplementary material for: Follow-up of bowel endometriosis resections performed using the double circular stapler technique: A decade’s experience
Source: PLoS One. 2025 Apr 28;20(4):e0320138. doi: 10.1371/journal.pone.0320138 (PMC12036926; doi:10.1371/journal.pone.0320138)
Supplement: Attachment — Submitted filename: rebuttal letter (reviews 2023).docx [file pone.0320138.s002.docx]

**REBUTTAL LETTER (PREVIOUS ARTICLE SUBMITTED - 2023)**

PONE-D-23-16370

Long-term follow-up of bowel endometriosis resections performed using the Double Circular Stapler Technique: a decade's experience (*it was the former title before reviews*).

PLOS ONE

Dear Dr. Crispi Jr,

Thank you for submitting your manuscript to PLOS ONE. After careful consideration, we feel that it has merit but does not fully meet PLOS ONE’s publication criteria as it currently stands. Therefore, we invite you to submit a revised version of the manuscript that addresses the points raised during the review process.

Please carefully revise the manuscript according to reviewers’ suggestions.

Reviewers' comments:

Reviewer's Responses to Questions

**Comments to the Author**

1. Is the manuscript technically sound, and do the data support the conclusions?

The manuscript must describe a technically sound piece of scientific research with data that supports the conclusions. Experiments must have been conducted rigorously, with appropriate controls, replication, and sample sizes. The conclusions must be drawn appropriately based on the data presented.

Reviewer #1: Yes

Reviewer #2: Yes

2. Has the statistical analysis been performed appropriately and rigorously?

Reviewer #1: No

Reviewer #2: Yes

3. Have the authors made all data underlying the findings in their manuscript fully available?

The PLOS Data policy requires authors to make all data underlying the findings described in their manuscript fully available without restriction, with rare exception (please refer to the Data Availability Statement in the manuscript PDF file). The data should be provided as part of the manuscript or its supporting information, or deposited to a public repository. For example, in addition to summary statistics, the data points behind means, medians and variance measures should be available. If there are restrictions on publicly sharing data—e.g. participant privacy or use of data from a third party—those must be specified.

Reviewer #1: Yes

Reviewer #2: Yes

4. Is the manuscript presented in an intelligible fashion and written in standard English?

PLOS ONE does not copyedit accepted manuscripts, so the language in submitted articles must be clear, correct, and unambiguous. Any typographical or grammatical errors should be corrected at revision, so please note any specific errors here.

Reviewer #1: Yes

Reviewer #2: Yes

**Reviewer #1:** An interesting paper regarding this new surgical possibility in cases of nodules, albeit extensive but not very infiltrative. Of course, as you have reported, this paper has different limitations that need, in the future, more articulated studies to confirm this new surgical approach.

Requests:

**Authors' response to Reviewer #1:**

First of all, we would like to thank you for the effort made to improve our manuscript. At this moment, we feel very happy about it. Undoubtedly, the more rigorous the review, the higher the quality of the publication. All issues raised (suggestions and criticisms) were carefully considered.

Our answers are presented below, point by point.

We recommend a re-evaluation of the tables which are, at times, difficult to use.

Yes. We fully agree. Unfortunately, the tables have become difficult to use. Actually, as this is an essentially descriptive study, we started from two premises: (1) Unlike figures, photos and graphs (which must present a visual message that is easy to be understood), a table was made to be consulted, that is, it has the function of informing details, which can be accessed if the reader wants/needs to find such information; (2) Our idea is that the reader will be able to reconstruct the cases individually in his/her imagination. For these reasons, we chose to gather the maximum amount of information in the smallest number of tables. Besides, we chose that each table fit on an A4 sheet, when printed on paper. After discussing the tables, we concluded that (despite being difficult) they achieved these objectives.

The statistical method used is not very clear, a re-evaluation or a better transcription of this section is requested if possible.

The section "Statistics" has been rewritten. The new version reads as follows (page 14):

" *As a descriptive study, this case series report was mathematically limited to calculating the probability of occurrence of some events. For this purpose, the calculation of absolute risk and confidence intervals were performed using IBM SPSS Statistics Version 29.0.0.0 (IBM Corp., Ar-monk, NY, USA). Although possible in theory, no statistical test comparing before and after out-comes was performed because the duration of follow-up was not standardized.*"

It is possible to have intraoperative images to better explain the surgical technique.

If this is a question (?), the answer to this question would be YES. In fact, this was our initial idea. However, presenting details of the 43 cases individually has always been the main goal of this study (in our opinion, an original approach). As a consequence, the manuscript became too long. Therefore, we chose to use one figure with 14 drawings, which we believe are easier to understand. After your suggestion, we have considered adding a video to be made available to PLosOne readers as supplementary material (if this is possible).

If it is possible to have a general summary table of the characteristics of the patients and the interventions associated with them.

Table 1 presents individual characteristics (both prior to surgery and at follow-up); Table 2 presents some details of the interventions (individually). Unfortunately, we were not able to understand this suggestion fully. Could you be more specific?

It is asked if possible how they find the possible urinary and colorectal complications compared to the newer evaluations as in Ianieri MM et al.2022, O'Brien et al. 2023.
and what difference, if any, compared to patients with endometriosis studied for a similar follow-up timeline as in Mabrouk et al. 2019.

First, we would like to present some specific comments about each reference:

[1] Ianieri MM et al.2022 - The study by Ianieri MM et al.2022 evaluated the functional outcomes of nerve-sparing surgery for endometriosis with or without posterolateral parametrectomy, using the BFLUTS tool to assess urinary symptoms. We would have liked the study to have reported partial BFLUTS scores (BFLUTS voiding, BFLUTS filling, BFLUTS - LUTS), so that the assessment of the most common urinary symptoms of endometriosis would not be "polluted" by data from the domains BFLUTS-incontinence, BFLUTS-sexual function and BFLUTS-QoL. Another important fact, which makes it difficult to compare results, is that the study by Ianieri MM et al.2022 excluded women with gross involvement of hypogastric nerves or inferior pelvic plexus during surgical dissection (which was not the case in our study). Therefore, we are dealing with a population with a different disease pattern and with a different outcome expectation. Given that the assessment of urinary symptoms was performed with different tools, different segment times (the study by Ianieri MM et al. 2022 focused on the outcome at 3 months) and in a different population, we consider the comparison of results inappropriate. [Ianieri MM, Raimondo D, Rosati A, et al. Impact of nerve-sparing posterolateral parametrial excision for deep infiltrating endometriosis on postoperative bowel, urinary, and sexual function. Int J Gynaecol Obstet. 2022;159(1):152-159. doi:10.1002/ijgo.14089]

[2] O'Brien et al. 2023 - The meta-analysis by O'Brien et al.2023 did not assess urinary complications and did not report the frequency of complications in patients undergoing intestinal resection; the study reported only the respective ORs of comparisons between surgical techniques. Their study included patients submitted to rectosigmoid resection due endometriosis and suggest that the functional outcomes are similar between conservative and formal resection; the complication rate did not differ between the discoid excision and formal resection. [O'Brien L, Morarasu S, Morarasu BC, et al. Conservative surgery versus colorectal resection for endometriosis with rectal involvement: a systematic review and meta-analysis of surgical and long-term outcomes. Int J Colorectal Dis. 2023;38(1):55. Published 2023 Feb 27. doi:10.1007/s00384-023-04352-6]

[3] Mabrouk et al. 2019 - A large and very interesting retrospective cohort study. It focused on patients with preoperative intermediate risk of segmental bowel resection, and the data suggest that conservative surgery is preferred over radical surgery in these patients. [Mabrouk M, Raimondo D, Altieri M, et al. Surgical, Clinical, and Functional Outcomes in Patients with Rectosigmoid Endometriosis in the Gray Zone: 13-Year Long-Term Follow-up. J Minim Invasive Gynecol. 2019;26(6):1110-1116. doi:10.1016/j.jmig.2018.08.031]

We inserted the paragraph below in the INTRODUCTION (topic: Complications in bowel resection – page 5):

*"A meta-analysis of patients who underwent rectosigmoid resection for endometriosis found that the functional outcomes were comparable between conservative and segmental resection, and that the complication rate did not differ between discoid excision and segmental resection (O'Brien L et al., 2023). However, observational data comparing long-term surgical, clinical, and functional outcomes between conservative and radical surgery in patients with rectosigmoid endometriosis suggest that conservative surgery (shaving, discoid excision) is preferred over radical surgery (segmental resection) in patients with rectosigmoid endometriosis (Mabrouk et al., 2019). Actually, further to the direct impact of the intestinal resection technique, the infiltration of the lateral parametrium should be brought into consideration while evaluating functional outcomes (Ianieri et al., 2022)."*

>>>>>>>>>>>>>>>>>>>>>>>>>>>>>>>>>>>>>>>>>

**Reviewer #2**: Dear Authors,

congratulations for your interesting manuscript which provides a good insight of the DCSR technique for colorectal DE.
However, I suggest you some amendments on order to obtain a greater version of your paper. I also suggest you reducing autocitations throughout the text.

**Authors' response to Reviewer #2:**

First of all, we would like to thank you for the effort made to improve our manuscript. At this moment, we feel very happy about it. Undoubtedly, the more rigorous the review, the higher the quality of the publication. All issues raised (suggestions and criticisms) were carefully considered. This case series report addresses a very specific topic (Double Circular Stapler Technique), which consists of a technique to be used in special situations. Most considerations presented in this manuscript were based on the experience of a multidisciplinary team when observing different outcomes in a retrospective cohort. Therefore, we believe it is important that some of the group's publications concerning endometriosis surgery are not omitted in this publication so that the reader realizes that the opinions presented are the result of more than a decade of surgical practice and academic work.

Our answers are presented below, point by point.

1- do you think the term "long-term" is adequately used in the title, according to the follow up range you report? I would rephrase it, according also to the terms you used throughout the text (short and long term).

The title was changed (page 1):

"*Follow-up of bowel endometriosis resections performed using the Double Circular Stapler Technique: a decade's experience".*

2- Please remove the term "only" throughout the abstract, it is a too subjective word when speaking of results.

We think this detail was a very pertinent suggestion. Thank you very much for your careful attention. We removed the term "only" throughout the abstract.

3- Insert percentages for this sentence "Most patients showed improvement in pain scores".

Perhaps, this has been the hardest issue. Actually, we evaluated 8 different types of endometriosis-related pain, which are presented in Tables 3 (urinary outcomes) and 4. All patients were thoroughly evaluated for both menstrual (M) and nonmenstrual (NM) endometriosis-related symptoms: M-Dysuria, NM-Dysuria, M-Dyschezia NM-Dyschezia, Dysmenorrhea, Deep Dyspareunia and cyclic low back pain. Pain-related symptoms were measured on an 11-point (0-10) self-reported numerical rating scale (NRS). Patients were instructed to report the intensity of pain considering the last 6 months.

Briefly,

M-Dysuria: 100% achieved a favorable outcome; NM-Dysuria: 100% achieved a favorable outcome; M-Dyschezia: 2 cases (4.7%) did not achieve a favorable outcome; NM-Dyschezia: 4 cases (9.3%) did not achieve a favorable outcome; Dysmenorrhea: 3 cases (7.0%) did not achieve a favorable outcome; Dyspareunia: 5 cases (11.6%) did not achieve a favorable outcome; CLBPain: 3 cases (7.0%) did not achieve a favorable outcome; PPain: 5 cases (11.6%) did not achieve a favorable outcome.

We could develop and include these details. But honestly, instead of presenting pain improvements in a percentage way, we would like to explore the cases only individually. The reason for this is the fact that the follow-up times are not the same, that is, other factors (in addition to the surgery) may have influenced the improvement or worsening of these symptoms over time. Therefore, we have rewritten the abstract in order to meet your requests about pain changes. The sentence below was inserted in the ABSTRACT (page 2) and in the RESULTS (Topic: Pain – page 24):

*"At the most recent follow-up on dysuria, dyschezia, dysmenorrhea, dyspareunia and cyclic low back pain, 88 to 100% of women showed any favorable response (improvements ≥3 points in symptomatic women or asymptomatic women who remained pain-free)."*

Besides, the paragraph below was inserted in the METHODS (Topic: Pelvic symptoms assessment – page 9):

*"The value of the minimal clinically important differences to assess variations in pain scores could be considered as low as 10% (10 mm on a 100 mm visual scale) (Bourdel et al., 2014). However, this study adopted a more conservative threshold to define a relevant change in pain after surgery; only those variations greater than 3 points were considered clinically relevant (Gerlinger et al., 2010). Thus, for each of the pain symptoms assessed, symptomatic women who reported an improvement in pain severity by 3 or more points or asymptomatic women who remained pain-free after surgery (score = 0) were labeled as having achieved a favorable response."*

*[Bourdel N, Alves J, Pickering G, Ramilo I, Roman H, Canis M. Systematic review of endometriosis pain assessment: how to choose a scale? Hum Reprod Update. 2015 Jan-Feb;21(1):136-52. doi: 10.1093/humupd/dmu046.]*

*[Gerlinger C, Schumacher U, Faustmann T, Colligs A, Schmitz H, Seitz C. Defining a minimal clinically important difference for endometriosis-associated pelvic pain measured on a visual analog scale: analyses of two placebo-controlled, randomized trials. Health Qual Life Outcomes. 2010 Nov 24;8:138. doi: 10.1186/1477-7525-8-138.]*

4- Recruitment time stops in July 2021: why did you stop so early and do not have more recent data?

All cases were included. The number of surgeries was significantly reduced in 2021/2022 due to the serious health problems faced by Prof. Crispi (*in memoriam*).

5- You do say you used validated tools to assess bowel symptoms (see abstract and methods). However these tools (reference 43-44, line 210-211) were published years later than the time at which most of the patients were included and assessed for the study. Please explain.

In fact, this comment (number 5) suggests the existence of a mistake. However, we think the explanation for this "apparent paradox" has been in the 4th paragraph of the section "Database and preplanned data collection" (page 9).

"...*To have the most current outcome information possible, all patients were contacted between July 2020 and February 2021 to complete the standardized abstraction forms specially designed for this study, which includes an assessment instrument which focused on pelvic pain, lower urinary tract symptoms, bowel function and reproductive outcomes.*"

6- Please specify if you assessed intra and/or postoperative complications (see abstract, methods section; see methods later on).

We included the terms "intra and postoperative":

(1) in the abstract;

(2) in two sections of the Methods ["Study design" (page 7) and "Surgery complications" (page 14)].

7- please rephrase the discussion/conclusion section of the abstract, particularly the sentence regarding the distance from the anal verge. You did not evaluate the distance with such detailed and conclusive data, and some of the data were missing, so giving such straight conclusions is a little too much.

We fully agree. We rephrased it (page 2): “Preliminary results were encouraging in the past. The current assessment including long-term follow-up supports DCST for colorectal DE as a feasible, useful, and safe strategy for avoiding segmental colorectal resection when appropriately indicated and properly performed.”

8- introduction: remove citation [8], pelvic floor muscle are not a so common site of disease and here you are generally speaking of endometriosis locations.

If the deletion of this reference [citation 8] is an imperative condition for the manuscript to be accepted for publication, we can do so. However, we would like to draw attention to the existence of the word "even" [an adverb used to refer to something surprising, unexpected, unusual or extreme], which we have used precisely to emphasize the breadth of possibilities of endometriosis presentation and the level complexity of the cases included in this cohort.

9- Line 79: "a circular stapler to transect a linear rectal stapled line" please rephrase to make it more clear.

We fully agree. We rephrased it. The new version reads as follows (page 4):

"*...Performing a colorectal anastomosis after a segmental resection using staplers offers some advantages over a traditional hand-sewn anastomosis: 1) there is significantly less contamination, 2) the anastomosis is technically easier to perform, and 3) bowel segments of different diameters can be easily anastomosed."*

10- I suggest you enriching the introduction section by reinforcing the concept that discoid and shaving techniques are safer and less invasive for colorectal DE, when feasible (10.1016/j.jmig.2018.08.031; 10.1007/s00384-023-04352-6) in order to give more importance to your study results and the technique you propose.

Thank you very much for one more valuable contribution. The references were included.

*[Mabrouk M, Raimondo D, Altieri M, et al. Surgical, Clinical, and Functional Outcomes in Patients with Rectosigmoid Endometriosis in the Gray Zone: 13-Year Long-Term Follow-up. J Minim Invasive Gynecol. 2019;26(6):1110-1116. doi:10.1016/j.jmig.2018.08.031]*

*[O'Brien L, Morarasu S, Morarasu BC, et al. Conservative surgery versus colorectal resection for endometriosis with rectal involvement: a systematic review and meta-analysis of surgical and long-term outcomes. Int J Colorectal Dis. 2023;38(1):55. Published 2023 Feb 27. doi:10.1007/s00384-023-04352-6]*

We inserted the sentences below in the INTRODUCTION (topic: Complications in bowel resection – page 5):

*"A meta-analysis of patients who underwent rectosigmoid resection for endometriosis found that the functional outcomes were comparable between conservative and segmental resection, and that the complication rate did not differ between discoid excision and segmental resection (O'Brien L et al., 2023). However, observational data comparing long-term surgical, clinical, and functional outcomes between conservative and radical surgery in patients with rectosigmoid endometriosis suggest that conservative surgery (shaving, discoid excision) is preferred over radical surgery (segmental resection) in patients with rectosigmoid endometriosis (Mabrouk et al., 2019). Actually, further to the direct impact of the intestinal resection technique, the infiltration of the lateral parametrium should be brought into consideration while evaluating functional outcomes (Ianieri et al., 2022)."*

11- Introduction: I would add a section speaking of anatomically-based nerve-sparing techniques and surgery for colorectal DE surgery here (https://doi.org/10.1002/ca.24059). I would also discuss it later in the discussion section as well as in the section speaking of the distance from the anal verge and the choice of the best bowel resection technique.

Thank you very much for the opportunity to provide more details. To fulfill your request, we added a specific section ("*Implications of the anatomy in nerve-sparing techniques for colorectal endometriosis surgery*") in INTRODUCTION (pages 3 and 4), which reads as follows:

[Obs. This reference was also included in the "Choosing the bowel resection technique" section (DISCUSSION)]

*"When dealing with intestinal endometriosis, pelvic nerves preservation begins with the choice of a conservative resection technique (shaving or discoid). Thus, whenever possible, less invasive procedures should be applied to facilitate preservation of the vascularization and the innervation of the bowel wall [Centini et al., 2023]. Conservative techniques for bowel resections (shaving and discoid) provide the advantage of minimizing manipulation of the mesorectal tissue, responsible for the vascularization and innervation. Therefore, segmental resection may ultimately be reserved for advanced lesions responsible for major stenosis or for several cases of multiple nodules infiltrating the rectosigmoid junction or sigmoid colon, when conservative resections are not surgically possible [Donnez et al., 2017].*

*In relation to the innervation of the pelvis, the superior hypogastric plexus ends up to the two hypogastric nerves, which travel along the lateral walls of the rectum before meeting the pelvic splanchnic nerves (S2-S4 roots) to form the inferior hypogastric plexuses. Thus, constituted by a complex tangle of nerves, the inferior hypogastric plexuses contain both parasympathetic and sympathetic fibers. Currently, nerve-sparing surgeries have been systematized with the thought that recognizing nerve structures is necessary for their preservation at the same time as understanding that surgical manipulation is not free risk and unnecessary dissections must be avoided [Aleksandrov A et al., 2022; Lemos N et al., 2015]. Some studies suggest the middle rectal artery is an anatomical landmark to discriminate whether to perform a segmental resection of the rectum, without the need to state a recommended distance from the anal margin [Centini et al., 2023]. Then, when the surgeon is developing the pararectal space, it is also important to identify the middle rectal artery.*

*Summarily, considering the propositions above, the first step of the systematization in this series has been to identify the ureters bilaterally at the level of crossing with the iliac vessels, followed by the development of the medial pararectal spaces. Secondly, the development of the pararectal space and identification of the hypogastric nerves should be guaranteed. Finally, with the anatomical landmarks recognized, it's possible to decide on the limit of nerve sparing and the radicality of surgery."*

*[Centini G, Labanca L, Giorgi M, et al. The implications of the anatomy of the nerves and vessels in the treatment of rectosigmoid endometriosis [published online ahead of print, 2023 May 11]. Clin Anat. 2023;10.1002/ca.24059. doi:10.1002/ca.24059.]*

*[Donnez O, Roman H. Choosing the right surgical technique for deep endometriosis: shaving, disc excision, or bowel resection? Fertil Steril. 2017 Dec;108(6):931-942. doi: 10.1016/j.fertnstert.2017.09.006. PMID: 29202966.]*

*[Lemos N, Souza C, Marques RM, Kamergorodsky G, Schor E, Girão MJ. Laparoscopic anatomy of the autonomic nerves of the pelvis and the concept of nerve-sparing surgery by direct visualization of autonomic nerve bundles. Fertil Steril. 2015 Nov;104(5):e11-2. doi: 10.1016/j.fertnstert.2015.07.1138. Epub 2015 Aug 8. PMID: 26260200.]*

*[Aleksandrov A, Smith AV, Botchorishvili R, Rabischong B. How to dissect the pelvic nerves: from microanatomy to surgical rules. An evidence-based clinical review. Facts Views Vis Obgyn. 2022 Mar;14(1):17-29. doi: 10.52054/FVVO.14.1.011. PMID: 35373544; PMCID: PMC9612858.]*

*Obs. The sentence below was added to the "The Double Circular Stapler Technique" section (INTRODUCTION – page 6):*

*"In sum, the DCST is about avoiding a segmental resection in order to reduce the risk of functional impair-ments potentially caused by surgery [Oliveira MA et al., 2014]."*

12- Line 125-126: please provide references for circumference and lumen bowel obstruction.

Sorry. There was a mistake. We corrected it and added some details (one more reference). The new version reads as follows:

*"* *Circumferential nodules or infiltrations responsible for advanced rectal stenosis can rarely be managed by shaving or nodule excision; in other cases, rectal defect resulting from full-thickness excision is so great that correct suture of the digestive wall is impossible, making colo-rectal resection inevitable [44]. Some approximate landmarks can be used as selection criteria for the DCST: (1) single colorectal lesion is between 2 and 5 cm in length, (2) the lesion affects no more than 40% of the circumference, and (3) the lesion does not obstruct more than 25% of the intestinal lumen [45,46].."*

*[Roman H, Vassilieff M, Tuech JJ, Huet E, Savoye G, Marpeau L, Puscasiu L. Postoperative digestive function after radical versus conservative surgical philosophy for deep endometriosis infiltrating the rectum. Fertil Steril. 2013 May;99(6):1695-704. doi: 10.1016/j.fertnstert.2013.01.131. Epub 2013 Mar 7. PMID: 23465818.]*

13- Line 186-198: this section is not clear to the reader. Could you please rephrase it? It seems like you had different kind of patients evaluations prior to January 2018 and this would affect the results.

We fully agree. Actually, aiming to present the methodology as completely as possible, we used unnecessary details, which made the text boring and confusing. [Thanks for your careful review!] The 2 paragraphs were rephrased (now as a single paragraph); the essential contents were retained (page 9).

The new version reads as follows:

*"* *Data from both preoperative and immediate postoperative periods (up to 40 days) were obtained by retrospective chart abstraction, whereas data from the surgeries were prospectively collected into a more structured and thus standardized database, including a review of videos of the surgeries. To have the most current outcome information possible, all patients were contacted by telephone, videoconferencing and email between July 2020 and February 2021 to complete the standardized abstraction forms specially designed for this study - an assessment instrument which focused on pelvic pain, lower urinary tract symptoms, bowel function and reproductive out-comes. The retrospective chart abstraction was performed by two experienced nurses with post-graduate training in clinical research. All data were double-checked to ensure maximum accuracy. Finally, the data were abstracted from the updated medical records into a database in February 2021."*

14- Line 201: provide reference for NRS pain evaluation.

One specific reference was included and the sentence was rephrased (page 9).

*[Williamson A, Hoggart B. Pain: a review of three commonly used pain rating scales. J Clin Nurs. 2005;14(7):798-804.]*

*"The severity of the pain was verbally assessed on a self-reported 11-point (0 to 10) numeric rating scale [Williamson et al., 2005]."*

15- I would add this reference to lateral compartment section: https://doi.org/10.1002/ijgo.14089.

Ianieri MM et al.2022 - The study by Ianieri MM et al.2022 evaluated the functional outcomes of nerve-sparing surgery for endometriosis with or without posterolateral parametrectomy.

*[Ianieri MM, Raimondo D, Rosati A, et al. Impact of nerve-sparing posterolateral parametrial excision for deep infiltrating endometriosis on postoperative bowel, urinary, and sexual function. Int J Gynaecol Obstet. 2022;159(1):152-159. doi:10.1002/ijgo.14089].*

The second paragraph of the section "The lateral compartment (parametrial region)" was rephrased (it was divided into two paragraphs) in order to include some contents from this reference (page 10).

*"The parametrium is a richly innervated cellular connective tissue located between the layers of the broad ligament that is commonly infiltrated by DE [3], which can be considered the "neurological electrical unit" of the pelvic viscera; the excision of posterolateral parametrial endometriosis seems to be associated with a higher risk of postoperative dyspareunia and sexual dysfunction compared with women without involvement of the parametria by endometriosis (Ianieri et al., 2022).*

*In this case series, we carefully reviewed resections performed in the lateral compartments, as autonomic nerves that must be protected as much as possible during surgery permeate them. In our detailed description of each of the 43 cases, we employ the 1997 Revision of the American Society for Reproductive Medicine classification of endometriosis (rASRM) [47]."*

16- Line 240-245: please provide references for patients preoperative preparation.

We understand that this suggestion (number 16) relates specifically to bowel preparation.

Preoperative bowel preparation is used to remove feces from the bowel lumen and reduce bacterial colonization. There has been no statistically significant evidence that patients benefit from mechanical bowel preparation, but the few studies focused on rectal surgery suggested that mechanical bowel preparation could be used selectively, even though no significant benefits have been found (Güenaga et al., 2011). Considering the lack of strong evidence on preoperative bowel preparation (Güenaga et al., 2011), RCTs studies are still needed to determine the most effective approach of preoperative bowel preparation for preventing complications in elective colorectal surgery (Willis et al., 2023).

In this series, the strategies adopted to prepare the patient were determined by the surgeon in charge (Dr CPC) based not only on scientific publications, but also on the professional experience of his multidisciplinary team.

*[Güenaga KF, Matos D, Wille‐Jørgensen P. Mechanical bowel preparation for elective colorectal surgery. Cochrane Database of Systematic Reviews 2011, Issue 9. Art. No.: CD001544. DOI: 10.1002/14651858.CD001544.pub4. Accessed 14 August 2023.]*

*[Willis MA, Toews I, Soltau SL, Kalff JC, Meerpohl JJ, Vilz TO. Preoperative combined mechanical and oral antibiotic bowel preparation for preventing complications in elective colorectal surgery. Cochrane Database Syst Rev. 2023;2(2):CD014909. Published 2023 Feb 7. doi:10.1002/14651858.CD014909.pub2.]*

This is a retrospective case series report and the procedures were described exactly as they were done. Therefore, 3 references were included and the paragraph was rewritten. The new version reads as follows:

" *Considering the lack of strong evidences on preoperative bowel preparation for prevent-ing complications in elective colorectal surgery [59, 60], the adopted strategies to prepare the patient were determined by the surgeon in charge (Dr C.P.C.) based not only on scientific publica-tions, but also on the professional experience of his multidisciplinary team.*"

17- Line 260-261: please provide references for these points "very extensive lesions and marked stenosis that do not allow the passage of the transanal stapler."

As these are practical rather than scientific points, this paragraph has been rewritten. The new version reads as follows (page 12):

*"For reasons inherent to surgical practice (which is explained by the size of the structures and instruments involved), there are some obvious situations in which DCST is not possible and segmental resection becomes necessary. They include multifocal rectosigmoid lesions (the resection of several segments would be necessary); very extensive lesions (DCST would not be able to remove the entire lesion), and marked stenosis that do not allow the passage of the transanal stapler. The current decision flowchart and eligibility criteria for the different surgical techniques for the treatment of bowel endometriosis are shown in Figure 1.*"

18- Statistics: enrich this section adequately.

The section "Statistics" has been rewritten. The new version reads as follows (page 14):

"*As a descriptive study, this case series report was mathematically limited to calculating the probability of occurrence of some events. For this purpose, the calculation of absolute risk and confidence intervals were performed using IBM SPSS Statistics Version 29.0.0.0 (IBM Corp., Armonk, NY, USA). Although possible in theory, no statistical test comparing before and after outcomes was performed because the duration of follow-up was not standardized*."

19- Although you did stated that you used a semi-qualitative method to report the results and we know that this is a case series, I would provide some percentages of the results as you did in the Pain section. This would help the reader to interprete the results and making them more discussable in the future. Please also add absolute number when reporting percentages.

There's a reason the "pain section" was presented in more detail. As stated at the beginning of the paragraph, the prevalences of different painful symptoms were quite heterogeneous. In addition, these symptoms were not presented as dichotomous variables (yes/no), but as numerical scales (ordinal variables), which were categorized and hierarchized: *"According to the instrument's scale, pain could be hierarchically categorized as none/mild (0-3), moderate/tolerable (4-6), or severe (7-10)."* (Lines 249/250/251).

As mentioned, our main strategy was to present the cases individually (raw data) rather than interpreting central measures or proportions, as the follow-up time was not standardized. However, we agree that some percentages of the results can be provided. Thus, besides the "Pain section" (pages 23/24), we had also presented some absolute numbers / percentages in the sections: "Main surgical findings" (page 18), "Immediate postoperative outcomes and surgical complications" (page 20), "Urinary findings" (page 23), "Bowel habits" (page 26) and "Reproductive outcome" (page 29) sections.

20- I would enrich the discussion section by highlighting even more the need for a nerve preserving surgical act (10.1111/ajo.12358) to prevent urinary complications in particular during colorectal endometriosis and the potential helpful procedures that can be used for the prevention of surgical complications such as the use ICG (10.1016/j.fertnstert.2018.02.122).

One sentence was included in the DISCUSSION section (topic Choosing the bowel resection technique –page 36). The new version reads as follows:

*"In addition to highlighting the need for a nerve preserving surgical act to prevent complications during colorectal endometriosis (Seracchioli et al., 2015), it is important to consider potential helpful procedures that can be adopted, such as the use of endovenous indocyanine green (Seracchioli et al., 2018) - strategy not used in this case series."*

*[Seracchioli R, Ferrini G, Montanari G, Raimondo D, Spagnolo E, Di Donato N. Does laparoscopic shaving for deep infiltrating endometriosis alter intestinal function? A prospective study. Aust N Z J Obstet Gynaecol. 2015;55(4):357-362. doi:10.1111/ajo.12358.]*

*[Seracchioli R, Raimondo D, Arena A, Zanello M, Mabrouk M. Clinical use of endovenous indocyanine green during rectosigmoid segmental resection for endometriosis. Fertil Steril. 2018;109(6):1135. doi:10.1016/j.fertnstert.2018.02.122.]*

6. PLOS authors have the option to publish the peer review history of their article ([what does this mean?](https://journals.plos.org/plosone/s/editorial-and-peer-review-process#loc-peer-review-history)). If published, this will include your full peer review and any attached files.


If you choose “no”, your identity will remain anonymous but your review may still be made public.


**Do you want your identity to be public for this peer review?** For information about this choice, including consent withdrawal, please see our [Privacy Policy](https://www.plos.org/privacy-policy).

Reviewer #1: No

Reviewer #2: No
